# Development of Intelligent Prefabs Using IoT Technology to Improve the Performance of Prefabricated Construction Projects

**DOI:** 10.3390/s19194131

**Published:** 2019-09-24

**Authors:** Linlin Zhao, Zhansheng Liu, Jasper Mbachu

**Affiliations:** 1 College of Architecture and Civil Engineering, Beijing University of Technology, Beijing 100124, China; linlinsuc@sina.com; 2 Faculty of Society & Design, Bond University, Gold Coast 4226, Australia; jmbachu@bond.edu.au

**Keywords:** prefabrication components, IoTs, RFID, LoRa, sensor networks, cloud-based BIM

## Abstract

Prefabrication (PC) projects have many advantages, such as cost and energy savings and waste reduction. However, some problems still exist that hamper the development of prefabrication projects. To improve PC project performance and advance innovation in construction, this study introduces an innovative method that incorporates Radio Frequency Identification (RFID) and Long Range (LoRa) technologies, sensor networks, the BIM model and cloud computing to automatically collect, analyze and display real-time information about PC components. It can locate PC components on a construction site and monitor their structural performance during the installation process. RFID technology and strain sensors were used to collect the required data on a construction site. All the data was transmitted to a server using LoRa technology. Then, the cloud-based Building Information Modelling (BIM) model of the project was developed to store and vividly present project information and real-time onsite data. Moreover, the cloud-based BIM model enables project team members to access the project information from anywhere by using mobile devices. The proposed system was tested on a real PC project to validate its effectiveness. The results indicate that the sensor network can provide reliable data via LoRa technology, and a PC component can be accurately located on site. Also, the monitoring data of structural performance for the PC component during the installation process is acceptable. The proposed method using innovative technologies can improve PC project performance and help industry professionals by providing sufficient required information.

## 1. Introduction

The generally recognized benefits of fast process, good quality, and a safe and clean working environment have made prefabrication construction (PC) a popular solution to various problems in the construction industry [1,2,3]. Despite the benefits of prefabrication, however, problems still exist. Stakeholders involved in PC projects face major challenges, such as installation error of PC components, inefficient information transmission among stakeholders, and lack of real-time information for visibility and traceability of PC components. Current PC projects exhibit poor data interoperability and a lack of ability to capture real-time data and information for visualizing and tracking PC components on-site [4,5]. In fact, it is a challenging task to efficiently identify, track and locate PC components on a dynamic construction site since it requires substantial amounts of data; in practice, this requires careful planning for data collection without interfering with on-site work. The current material tracking process is usually carried out manually, through paper-based approaches, which is time consuming, prone to error, and subjective in judgement [6]. A method that can automatically collect important onsite data is required.

Furthermore, the structural performance of PC components during the installation process is not predictable by design or simulations. Depending only on design models or simulations to assess the structural performance of PC components may lead to dangerous problems. This poses a significant challenge to structural safety, operation, and maintenance, which often besets the prefabrication industry. Continual monitoring the structural performance of PC components during the installation process to provide reliable data about their structural changes is required. Due to the dynamic nature of the construction site, continual monitoring is more challenging than operational monitoring. Current practices are based on a wired Structural Health Monitoring (SHM) system that usually disturbs on-site work and increases cost [7]. With the increased demand for a Structural Health Monitoring (SHM) system, an SHM system with wireless sensor network is required. A wireless sensor network eliminates the requirement of extensive wiring between sensors and data receivers, thereby allowing flexible configuration and lower installation cost [8].

In this study, a novel method based on cloud-based BIM and Internet of things (IoT) technologies is proposed. The proposed method incorporates cutting-edge technologies, such as Radio Frequency Identification (RFID) technology, wireless sensor networks, Long Range (LoRa) technology, Building Information Modelling (BIM), and cloud computing. The proposed method can obtain real-time data of a PC project on a dynamic construction site and share information via internet. The RFID sensors can help to locate the PC components on a construction site. Monitoring stress or strain levels in a structural component is an effective method for detecting the initiation and propagation of cracks since most concrete failures are due to cracks [8]. According to [9,10], changes in strain level are a critical parameter allowing detection of damage in a structure. Strain sensors can be adopted to provide strain levels of a PC component during the assembly process. All sensor data is wirelessly transmitted to an on-site computer server using LoRa technology. Then, the data is filtered and uploaded to a cloud-based data storage system. Next, the information about the PC components is vividly presented in the BIM model. A BIM model for exchanging information among project team members, checking prefabrication (PC) project information, and observing real-time information about the PC project can be developed. The BIM model can provide a 3D geometric model to help industry engineers visualize the locations of PC components in a virtual environment, which can provide more vivid information than 2D drawings. Moreover, project team members can access a project information anywhere using mobile devices, which improves their understanding of a project and facilitates their communication. In addition, the real-time information about the project on the construction site can be obtained. To validate the effectiveness of the proposed method, some tests have been conducted. First, the sensor network should be examined to assure its efficiency. Then, a field test to validate the capability of locating PC components and monitoring strain level of PC components during installation process, is conducted on a real PC project in Beijing.

The remainder of the paper is organized as follows: Section 2 presents a literature review about the previous works in related area. Section 3 describes the proposed method in detail. Section 4 presents a sensor network test and a field test of the proposed method applied to a real PC project. Section 5 discusses the results and concludes the paper.

## 2. Literature Review

### 2.1. Internet of Things (IoTs)

With the rapid development of information technology, IoT technologies (IoTs) have played a key role in almost every facet of various industries [11]. IoTs include sensors, actuators, electronic signalization, robots, and various other Internet-enabled physical devices that are provided for innovative and advanced smart applications in industries [12]. IoTs enable traceability and visibility of industrial processes and facilitates information sharing and big data analytics [4,13]. These innovative technologies also act as an engine that drives upgrading and transformation of the construction industry [14]. In this regard, there have been numerous attempts to practically apply such technologies. Reference [15] investigated the potential of applying RFID in the construction industry and identified benefits, including cost savings, quality improvement, and safety assurance. Reference [16] used RFID technology and wireless sensor networks to manage the construction supply chain. In addition, [17] proposed a framework for managing data and information of construction sites based on mobile computing. Reference [18] developed a BIM-based model that minimized energy consumption and improved building safety. Reference [19] used RFID technology for material tracking. Reference [20] integrated the BIM model with sensor technology to improve work-environment safety for construction workers. Similarly, [21] proposed a BIM model that benefitted safety control, productivity monitoring, and asset management in construction projects.

### 2.2. Positioning

Many technologies have been adopted for locating objects. For example, Global Positioning System (GPS) is capable of providing accurate outdoor position information, with centimeter accuracy [22]. However, GPS does not meet the requirements for sensing indoor locations. Various technologies for indoor positioning have been employed, including a GPS-based solution [23], Ultra-Wide Band (UWB) [24,25], and Wireless Local Area Network (WLAN) [26]. Signal measurements of indoor position include Received Signal Strength Indicator (RSSI), Time of Arrival (ToA), and Angle of Arrival (AoA) [27]. ToA can be used to measure the travel time of a signal between the sender and the receiver, but the signal velocity must be predefined [28]. The direction of signal transmission is typically considered when using AoA [29]. Some studies have been conducted that focused on the use of active RFID tags for indoor positioning [30] and outdoor localization supported by GPS [31].

Real-time positioning plays an important role in tracking the location of target objects. Recently, the number of studies focusing on the real-time positioning at construction sites has been increasing [32]. For example, [33] developed a Synchronized Position Attitude Navigation (SPAN) system incorporating an Inertial Measurement Unit (IMU) to improve GPS performance. [34] compared three indoor location technologies, including Wireless Local Area Network (WLAN), Radio Frequency Identification (RFID), and inertial measurement units (IMU) to locate building components that need to be repaired in order to support operations and maintenance. The findings suggest that RFID-based technology performs the best among these three. Reference [26] examined the feasibility of a Wi-Fi-based indoor positioning system to track the location of construction resources on site. The system has been validated on a shield tunnel construction site, with the results indicating the effectiveness and accuracy of the system. Reference [35] proposed a system integrating Fiber Bragg Grating (FBG) sensor networks with RFID-based labor-tracking technology to improve safety management in underground construction. Reference [36] presented a method for automatically collecting real-time positioning sensed data in order to improve on-site management and safety management.

### 2.3. LoRa

Wireless sensor networks play an important role in the Internet of Things (IoT), and many attempts have been made to achieve low-latency and energy-saving communication [37]. LoRa is a spread spectrum technique, based on the Chirp Spread Spectrum for long-range communication developed by [38]. LoRa stands for long range technology described in IEEE standard 802.15.4 [39]. Semtech’s long-range technology, called LoRa, allows data to be spread over both time and frequencies, which increases the range and robustness by increasing the sensitivity of the receiver [40,41,42]. The main LoRa parameters include Band Width (BW), Coding Rate (CR), and Spreading Factor (SF). The selection of the LoRa mode requires a trade-off between data rate, a longer range, and immunity to interference [41].

LoRa has become an innovative technology for IoT networks worldwide, which provides the industry with a diverse set of applications that range from tracking sensors for individuals with dementia to monitoring fertilizer levels in soil. LoRa technology enables smart IoT applications that solve some of the biggest problems, including energy management, pollution control, building and infrastructure efficiency and disaster prevention and evacuation. Several studies have been conducted to evaluate the performance of LoRa technology [43,44,45]. Reference [46] focused on LoRa scalability, while [47] concentrated on the spreading factor of Lora technology. Spreading factor is closely related to data transmission speed, covering a range of LoRa network and communication reliability. References [48,49] investigated configuration strategies to achieve optimal performance of a LoRa network.

Some studies have explored the application of LoRa technology in a wireless sensor network to automatically collect sensed data. For example, [50] suggested a wireless sensor network platform that would allow important and costly water networks to monitor utilities for the purpose of providing early warnings of leakage or deterioration. Study [51] used LoRa technology to gain real-time access to the telemetry data about behavior of fish in fish farms, in order to improve farm management. Study [52] described an IoT architecture incorporating LoRa technology for the monitoring of large-scale monumental structures. All these studies demonstrate the benefits of LoRa technology, such as long-range coverage, low energy consumption and cost saving. There are no studies that integrated LoRa technology with the BIM model for monitoring PC components during the installation process.

### 2.4. Structural Health Monitoring System

Currently, a variety of approaches and sensors have been used in Structural Health Monitoring (SHM) systems in civil infrastructures and high-rise buildings [53]. They can be appropriately categorized into two groups: local SHM methods and global SHM approaches [54]. The first method uses the propagation properties of an ultrasonic wave to detect the deficiency location, while the other utilizes vibrational characteristics of the projects to locate structural problems [55]. However, the global vibrational method is unable to measure small deficiencies, and the local propagational approach usually requires a large number of sensors in an extended network [54,55]. Study [55] used both wave propagation and vibrational methods to examine the severity of the physical deficiencies in different scenarios. The results indicate that both methods are effective and provide valuable findings. However, each method is costly, and a wired structure is not desirable. Study [56] used a vibrational method with piezoelectric patches embedded in concrete to detect structural problems. The authors of one study [57] used piezoelectric ceramic transducers to detect structural damage through the combination of electro-mechanical impedance and guided waves. Another study [58] employed active piezoelectric-based sensors for structural health monitoring based on electromechanical impedance technique. Reference [59] used a propagational method, with fiber optic sensors either embedded or surface-bonded onto the structural components, to detect structural problems by using time of flight information. Similarly, study [60] used both surface-mounted and embedded fiber optic sensors to monitor long-term global and local structure conditions of a high-rise building.

Conventionally, sensors are embedded into concrete, to monitor structural problems while the structure is being built. However, this method usually requires cables to transmit the sensed data to the data receivers. According to [61], the wired sensor structural health monitoring system is time-consuming and expensive. As addressed in [58], wireless sensing systems can be rapidly installed and uninstalled to develop a structural health monitoring system for temporary or emergency purpose at relative short time. Hence, some studies have been performed focusing on employing wireless sensor networks. For example, study [62] adopted a network of wireless sensor nodes to monitor vibration and wind levels of a cable-stayed bridge. Reference [63] used the collected data from accelerometers to establish a model for detecting physical deficiencies of a bridge. Study [64] employed a wireless sensor network of accelerometers, temperature sensors and foil strain gauges to monitor a highway bridge. Based on an extensive review of the existing literature, it seems clear that an effective Structural Health Monitor (SHM) system with reliable wireless communication is required.

Recently, non-contact visual measures have been used in structural health monitoring (SHM) systems. For example, [65] introduced a binocular vision method that provides a non-contact 3D deformation measurement for concrete-filled steel tubular columns. Moreover, [66] used correlation algorithm to improve the accuracy of multi-camera schemes.

### 2.5. Dynamic BIM

Currently, Building Information Modelling (BIM) has been widely used in the design and engineering phase to provide three-dimensional (3D) visualization model, project detailing, and data management. BIM is an effective method that allows users to evaluate scenarios of the construction process within a virtual environment. In general, Building Information Modelling (BIM) serves as an information hub that connects all project participants, but it is largely a static hub, in which real-time information cannot be automatically stored and updated to be incorporated into the model. In fact, BIM can be used to visualize sensor readings for resource location and tracking [67]. To mimic actual site conditions, BIM can incorporate Internet of Things technologies (IoTs) for automatic acquisition of real-time data. The IoTs provide a large amount of new data for BIM. Hence, BIM can provide a platform to organize and analyze the data. The integration of BIM and IoT may have many advantages in the Architectural, Engineering, and Construction (AEC) industry. Several studies have been conducted focusing on linking real-time data and BIM. For example, [68] provided a guidance that incorporates BIM and real-time data for an emergency response team to rapidly access navigation information of a facility under an emergency situation such as an earthquake, flooding and fire. Study [69] developed a BIM model to incorporate real-time data to detect energy faults in buildings for effectively maintaining building energy performance. Study [20] suggested a BIM model integrating wireless sensor technology to provide improved visualization for confined spaces on a construction site to reduce hazards. Study [70] developed a framework integrating a BIM model and IoT technology for in-use stage building to inform end-users about both the behavior of occupants and buildings. Study [71] suggested a generic approach for integrating BIM and real-time sensor data. Reference [72] developed a BIM model for facility management to aid facility managers in obtaining real-world environment information in a BIM model by incorporating real-time data from a Building Management System (BMS).

### 2.6. Gaps in Existing Literature

Given the uniqueness of PC components in prefabricated construction, item-level management is requested throughout the supply chain. Few studies have been conducted that focused on developing a management platform for a PC project, especially for the assembly process. Moreover, due to the special requirements of PC construction, information regarding the structural performance of PC components during installation process is required. Taking these gaps and requirements into account, efforts are needed to apply IoT technologies for the development of a platform for PC projects and to realize item-level PC component management and facilitate collaboration among project participants.

Four perspectives are significant for this research. First, RFID technology combined with triangulation technique was used to correctly locate PC components on construction site. Second, a wireless sensor network was devised to obtain real-time data regarding structural performance of PC components during installation process. Third, the sensed data were transmitted via LoRa technology. The LoRa technology is first used on a construction site to help in obtaining real-time onsite data. Finally, a cloud-based BIM model is developed to ease decision-making. The model enables project team members access real-time project information anywhere by using mobile devices. This innovative method involved advanced technologies and techniques can benefit both the literature and industry.

## 3. Proposed Method

**Figure 1:**
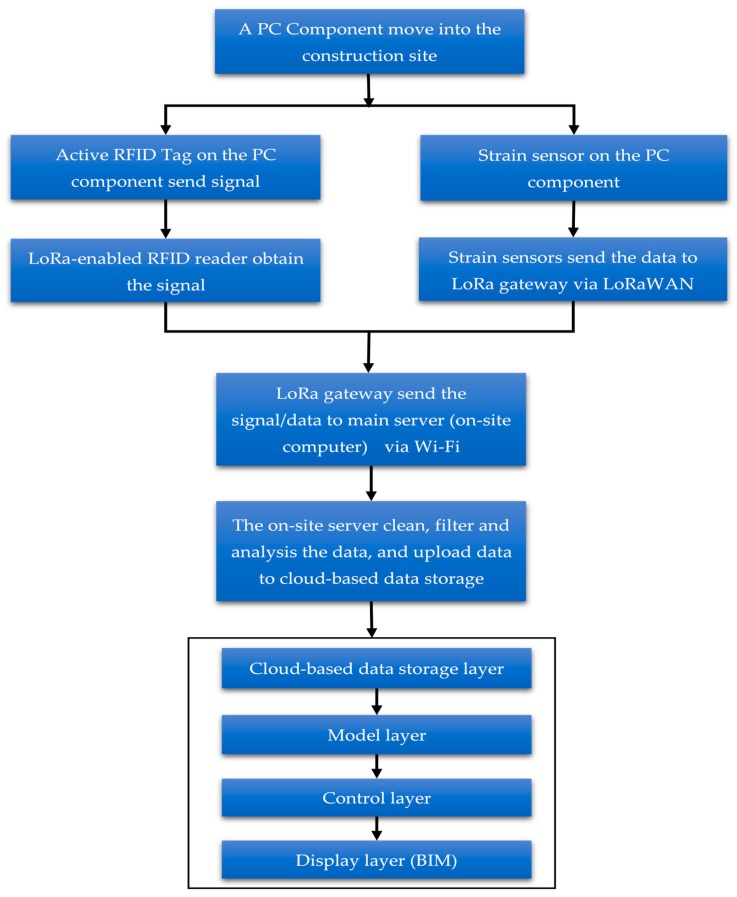
Flowchart illustrating the proposed method.

The proposed method that connects Internet of Things technologies (IoTs) to BIM tool develops an application programming system, which can be used to improve PC project performance. The proposed method includes four modules. The first module consists of Radio Frequency Identification (RFID) sensors and LoRa-enabled RFID readers on the construction site. This module is used to locate prefabrication (PC) components on the construction site. When a PC component is moving into a construction site, an active RFID sensor can broadcast signal that can be received by RFID readers on site. The second module is comprised of a wireless sensor network, which can be used to collect strain data of a PC component during the installation process. All the sensor data are transmitted using LoRa network technology. The sensor data is first transmitted to the LoRa gateway through Long Range Wide Area Network (LoRaWAN) protocol and then the data is sent from the gateway to an on-site computer server via Wi-Fi. The data is cleaned and filtered, and then uploaded to the cloud-based data storage. The fourth module is a cloud-based BIM model which includes four layers such as data storage layer, model layer, control layer and display layer. The model vividly presents project information and allows users to access real-time data. The details of the modules are illustrated in this section. The principle of the method is shown in Figure 1.

### 3.1. RFID Technology

Radio Frequency Identification (RFID) technology is used to identify the location of PC components on a construction site. LoRa technology has several advantages, but one of the most challenges is geolocation. An active RFID tag is placed on a PC component and LoRa-enabled RFID readers are located on the construction site. An active RFID tags has its own energy source, which allows them to broadcast its own signal to advertise its identity. The active RFID sensor can broadcast a signal that can be captured by a RFID reader on construction site. Based on RFID readers placed at three different locations, a triangulation technique can be used to analyze the signals to identify the location of a PC component. The main concept of the triangulation technique is based on the Received Signal Strength Indicator (RSSI). Signal strength is closely related to the distance between the sender and the receiver. Signal strength is attenuated during the propagation process. The distance between the sender and receiver can be estimated by using the degree of loss in signal strength. RSSI measurements use a path loss model to estimate distance. Hence, a set of tests were performed to explore the relationship between signal strength and distance. The relationship model was developed based on 1,980 laboratory test datasets. Each dataset included signals captured at a specific distance. Distances were varied at an interval of 20 cm. Linear regression analysis was performed to develop the relationship, as shown in Equation (1):

(1) r=−15.82RSSI+300

**Figure 2:**
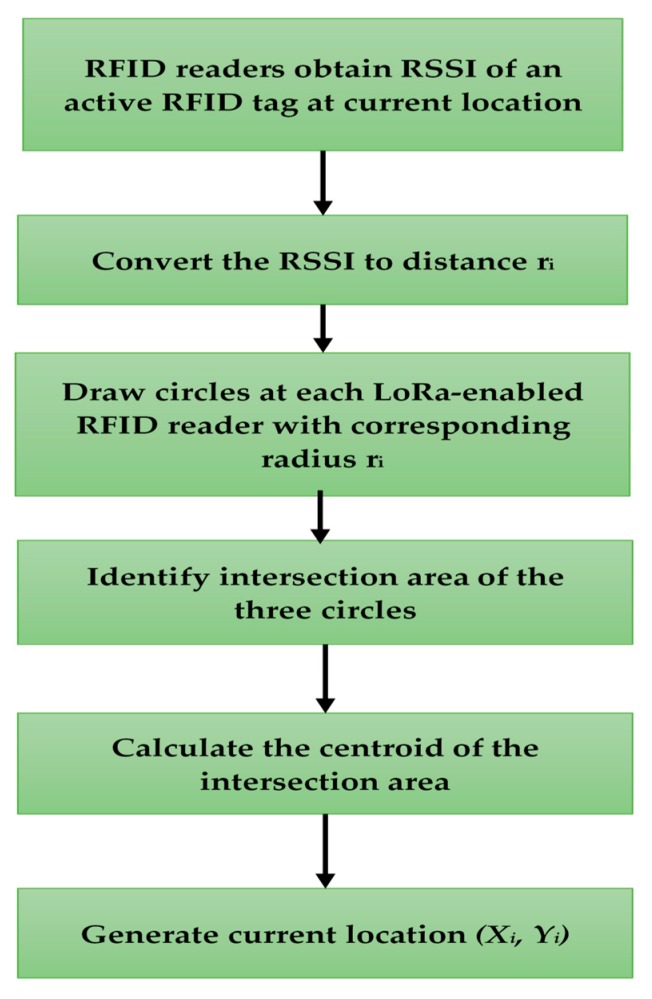
Flowchart illustrating the process of location identification using triangulation technique.

The signal captured by a reader can be converted into distance *r*, based on Equation (1), then a circle is generated. The center point of the circle is the location of the reader, the radius is the distance *r*. Three LoRa-enabled RFID readers are used as reference points with known positioning (marks) within a defined zone. The locations of three LoRa-enabled RFID readers are identified as $\left(x_{1},\;y_{1}\right),\;\left(x_{2},\;y_{2}\right),\;\mathrm{and}\;\left(x_{3},\;y_{3}\right)$ and the location of the PC component is identified as $\left(x_{i},\;y_{i}\right)$. The coordinates of the three locations are stored in the database. The three LoRa-enable RFID readers are located at the same height. Hence, there are three circles generated at the location of the three readers with respective radii. The centroid of the common area of the three circles is considered to be the location of the PC component $\left(x_{i},y_{i}\right)$. The flowchart of the location identification process is presented in Figure 2. 

**Figure 3:**
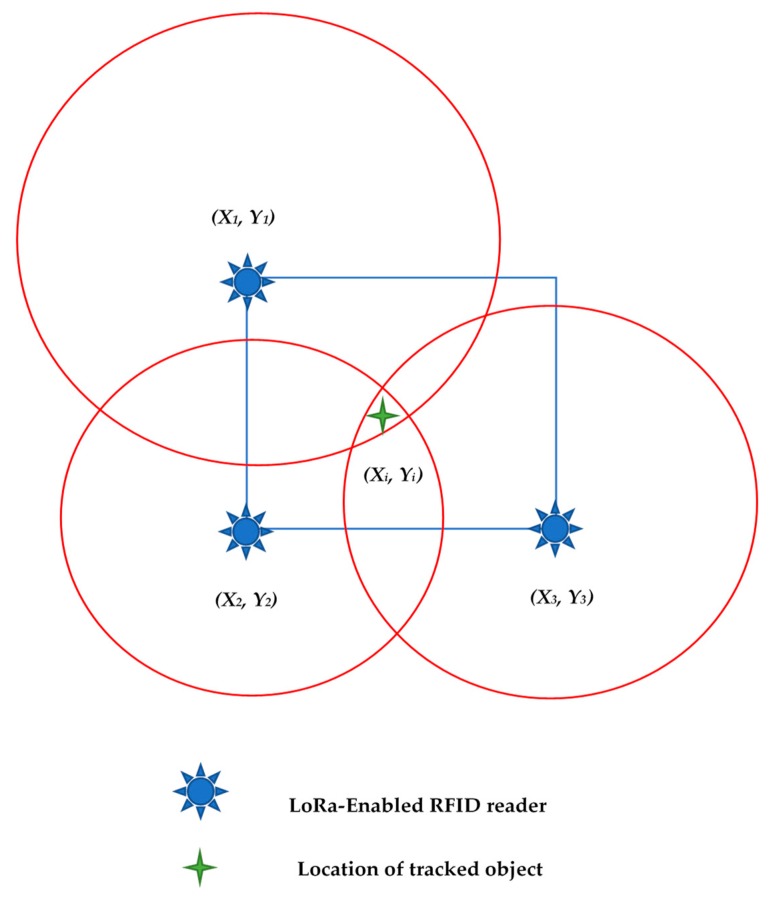
Sketch illustrating the triangulation technique.

The PC component moves to the next location on-site, and the same process is repeated. The generated location $\left(x_{i},y_{i}\right),$ with its corresponding time, is stored in the database for further analysis. The triangulation technique can be simply explained as an approach uses the intersection of the three generated circles to determine the position of a PC component. The main concept of triangulation methods is displayed in Figure 3.

### 3.2. Structural Performance Monitoring

Although prefabrication (PC) projects offer many advantages, they also have issues, such as structural integrity and secondary stress during the assembly process [73,74]. Monitoring structural performance of PC components during the installation process is essential to reduce dangerous risks. In fact, monitoring changes in strain is an effective way to evaluate structural performance [75,76]. Continuous real-time monitoring of dynamic strain in a structure can provide valuable information for structural assessments and inspections. The level of strain in PC components usually indicates the level of deflection in PC components. Strain information is important for assessing structural performance of PC buildings. Monitoring changes in strain on components during installation is necessary.

Traditional structural monitoring processes depend mainly on qualitative visual inspection [77]. A conventional structural health monitoring method, which uses traditional instruments and is taken randomly, is ineffective and inconvenient [78]. With the rapid development of sensors and information technology, adopting wireless sensor systems for structural health monitoring is inevitable. To this end, the present study proposes an intelligent system for monitoring the structural performance of PC components during the installation process. In this intelligent system, the strain sensors are attached at lifting points on a PC component. Then, strain level of a PC component can be monitored during the installation process. Strain data can be used in structural analysis for evaluating structural performance of a PC component. Real-time data obtained from sensors were directly transferred to an on-site server based on data transmission module. The server is an on-site computer with data processing software. Then, the clean data can be uploaded to a cloud-based server for further use.

### 3.3. Data Transmission

Connected distributed sensor networks for management, monitor and maintenance are the motor-driving of IoT [79]. One of the major challenges is offering reliable connectivity for distributed sensors and meeting the ultra-low-power consumption requirements. Designed for IoT communications, Long Range (LoRa) technology enables the connection between remote sensors and internet for delivery to analytics applications. Long Range (LoRa) technology enables hundreds of tags, sensors or actuators to connect to the network with low energy requirements. Moreover, transmission time is an important issue in the transfer of data. In this manner, clock synchronization should be achieved. The advantages of the LoRa system include: high accuracy, high speed, system integration, reduced noise levels, automatic, real-time data acquisition and web-based networking. The unique capability of LoRa technology is long-range, low-power wireless connectivity. Additionally, LoRa technology secures data transmission. Unfortunately, little is known about the merits of LoRa technology. This paper first introduces the application of LoRa technology in a realistic construction work environment.

**Figure 4:**
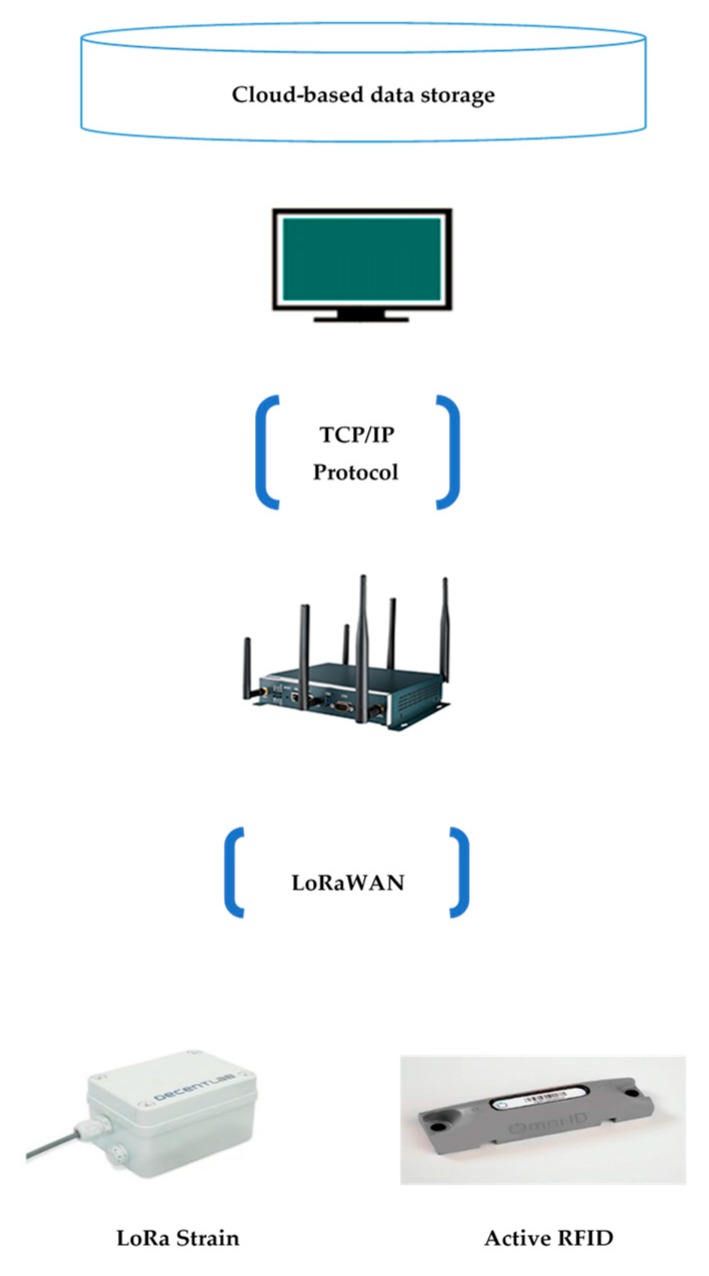
The architecture of the data transmission module.

**Figure 5:**
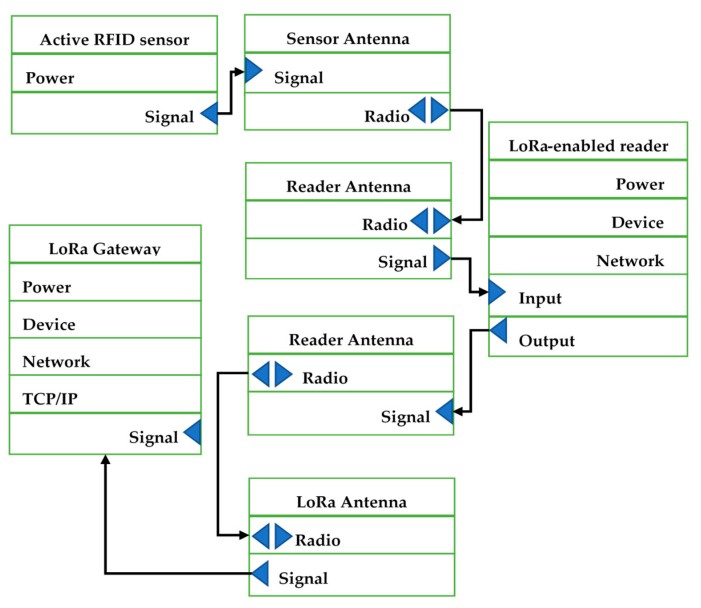
RFID location module connectivity.

Data captured by the end devices is transferred to the LoRa gateway that has an internet connection, which can send data to a network server. Long Range Wide Area Network (LoRaWAN) is a network protocol proposed by LoRaAlliance, which is designed to wirelessly connect things to the internet. The LoRaWAN open specification is a wide area networking protocol designed to wirelessly connect sensors to the Internet by leveraging the radio spectrum. A gateway can store the collected data and use Wi-Fi or Bluetooth to send data to a server, which is responsible for network management functions. For each data packet received, various information can be transferred to the gateway. The information contains packet information, such as packet type, source address, the packet’s RSSI, and data length; the radio information includes Band Width (BW), Coding Rate (CR), Spreading Factor (SF), and time of reception. The architecture of data transmission module is shown in Figure 4. The connectivity of an active RFID sensor and LoRa transmission network is displayed in Figure 5.

### 3.4. Data Management

A large amount of different types of data can be obtained during the monitoring process. Thus, it is necessary to build a powerful data management center. Moreover, for further use, data should be filtered to delete errors and noise. Abnormal signals, which are similar to noise, may impact the reliability of the data. For sensor data, the signal varies with time; the signal is stable and reliable, with no significant changes for a very short time. The collected data in a period of time should satisfy Equation (2) and should have a normal distribution within the interval [μ−2σ, μ+2σ] at a 95% confidence level. This indicates that 95% of data fall within the interval and only 5% of data are outside of the interval. This 5% of data are usually considered to be noise and need to be filtered out. An algorithm is used to filter the noisy data:

(2) $$x\in N\left(\mu ,\;\sigma^{2}\right),$$

Real-time data were collected, filtered, and stored for further use. Real-time data included the current location of a PC component, ID, name of the PC component, and trade. Data filtering was performed prior to data analysis. The final data output should be linked to Industry Foundation Classes (IFC) for data interoperability. An on-site host computer is located on site to manage all data collected from the sensors. The software program in the PC cleans the data by filtering noise, and ensures that all data are in the same format. Then, the data are sent to cloud-based data storage.

**Figure 6:**
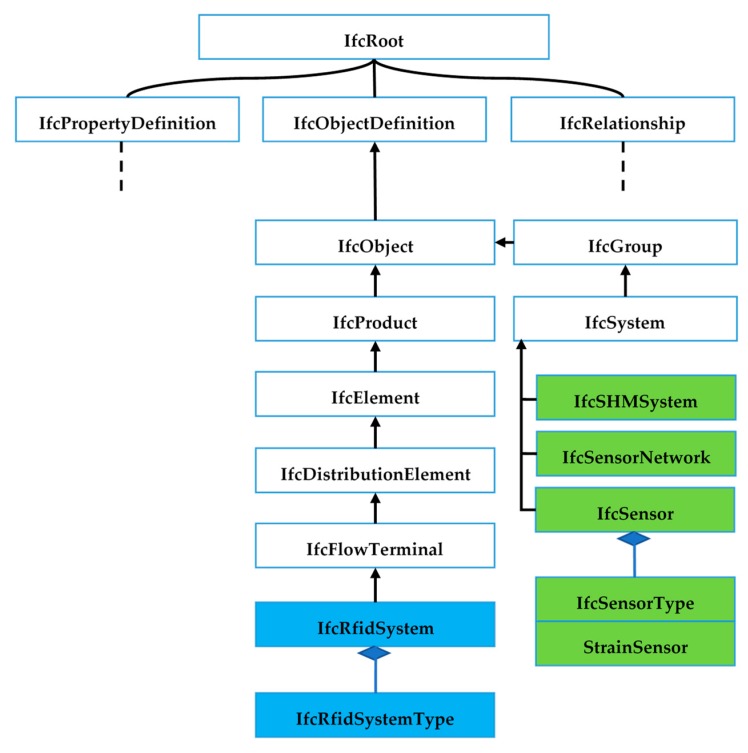
IFC hierarchy architecture and its extension with new entities (colored).

In recent studies, monitoring data is described by using Industry Foundation Classes (IFC), a standard data schema for a BIM-based model [80]. The IFC schema is extended. In total, four entities have been added to the IFC schema, including sensor nodes (IfcSensorNode), sensor networks (IfcSensorNetwork), Structural Health Monitoring (SHM) system (IfcSHMSystem) and RFID location module (IfcRfidSystem). The IFC schema and its extension entities are shown in Figure 6. The entities have been integrated into the existing IFC schema based on the semantic meaning of each entity. The extension of the IFC schema is in compliance with the EXPRESS data modelling language in ISO 10303-21: 2016 [81].

The hardware in RFID location module can be categorized into two major groups, including active RFID sensor and LoRa-enabled RFID reader. The RFID devices can be grouped into the IFC Electrical Domain schema, which is a part of the Domain Layer of the IFC schema [82]. The RFID location module can be defined as a subtype of IfcFlowTerminal which is a suitable subtype for the RFID elements since elements that produce data flow can be grouped under this category [83]. In the IFC schema, each RFID tag should be assigned to the PC component to which it is tagged. For example, if a prefab is tagged, the tag relates to the properties of a PC component (e.g., ID, name, trade) can be tracked. The main data consist of tag ID, location, and timestamp. Therefore, when a tag is read, all of the information about the PC component is available. Information about the PC component, such as on-site time, installation time, end time, and location can be collected, stored, and queried for further analysis. Similarly, the strain level of a PC component can also be tracked by using the monitoring data. The monitoring data include sensor ID, sensed value, sensed time, and other related information.

### 3.5. Cloud-Based BIM

Building Information Modelling (BIM), when combined with real-time on-site data, can provide an intelligent and robust system for managing installation of PC buildings. According to [84], combining real-time sensor data with BIM is a challenging task and is outside the scope of BIM’s functionality. Based on the suggestion of [85], using BIM in the monitoring process can improve the effectiveness of monitoring and decision-making. 

**Figure 7:**
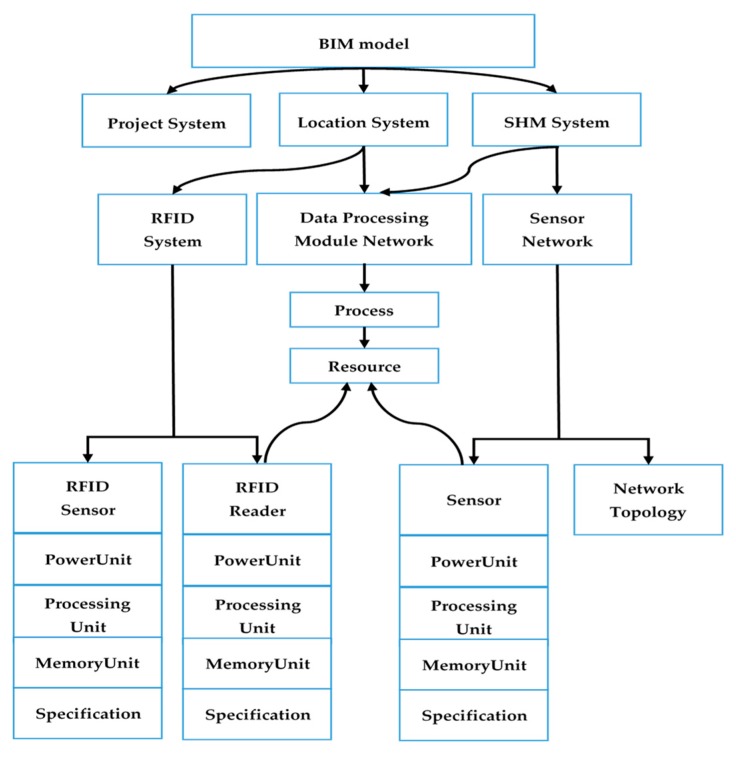
BIM model framework.

A BIM model include sufficient information include building information and real-time data can facilitate on-site management for a prefabrication (PC) project. The building information includes project information, such as building geometry and structural types. Real-time data of prefabrication (PC) components include the location of PC components on site and strain level of PC components during installation process. Figure 7 shows the BIM model for managing information of a prefabrication project and real-time data.

**Figure 8:**
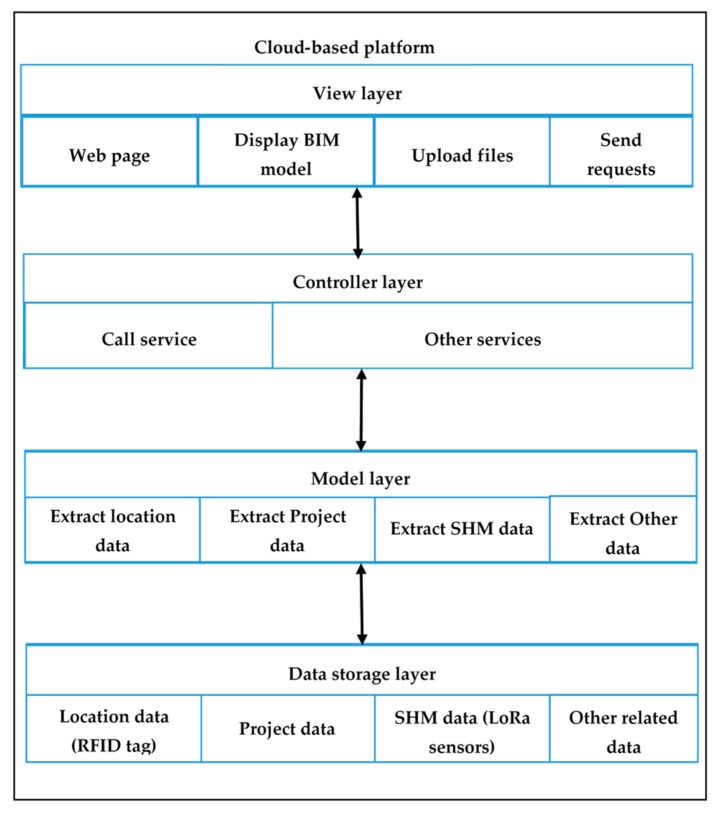
The four layers of the cloud-based BIM model.

The BIM model is built on a cloud-based platform. A cloud-based platform is comprised of a significant number of virtual servers, which are provided by cloud service vendors on a pay-per-use basis. The web page is a static website hosted on the cloud platform. A Model-View-Controller (MVC) architecture was used to develop the cloud-based BIM model with four layers, including the database layer, the model layer, the controller layer, and the view layer. The four layers of the BIM model is displayed in Figure 8. 

**Figure 9:**
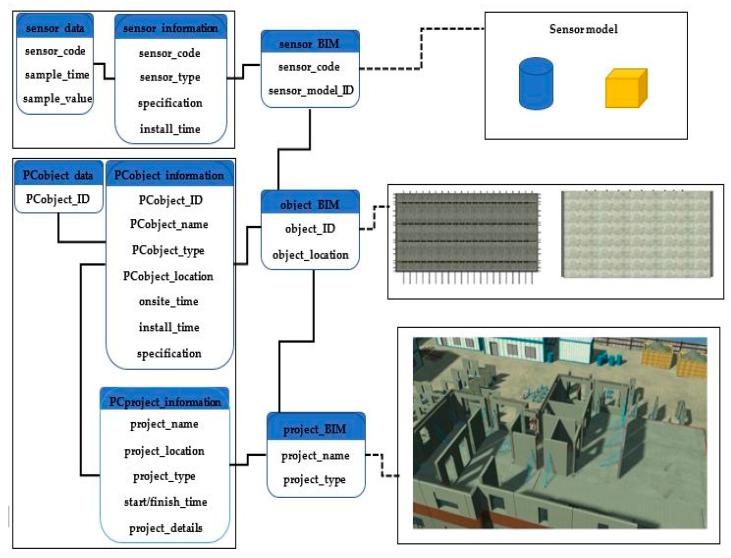
The information incorporated in BIM model.

The database layer is an information resource used to store data collected from the site, BIM data, and other related information. The model layer is used to extract information from the database layer, based on the commands from the controller layer. The controller layer manages the main logic between the model layer and view layer, which is responsible for providing appropriate responses to user requests. For example, when a user clicks a button to make a request, the controller layer invokes corresponding data from the model layer and the information presented on the view layer. The view layer is a user interface consisting of HTML components, which presents the model and information to the users. To develop the cloud-based BIM model, several development languages were used, including Java, JavaScript, SQL and jQuery. Users can log into the cloud-based BIM platform to obtain all the required information about PC components and project information. The information incorporated in the BIM model is illustrated in Figure 9. Through using cloud-based BIM, all information about the PC components is available on-line. This information can be shared across project teams.

## 4. Practical Application of the Proposed Method

**Figure 10:**
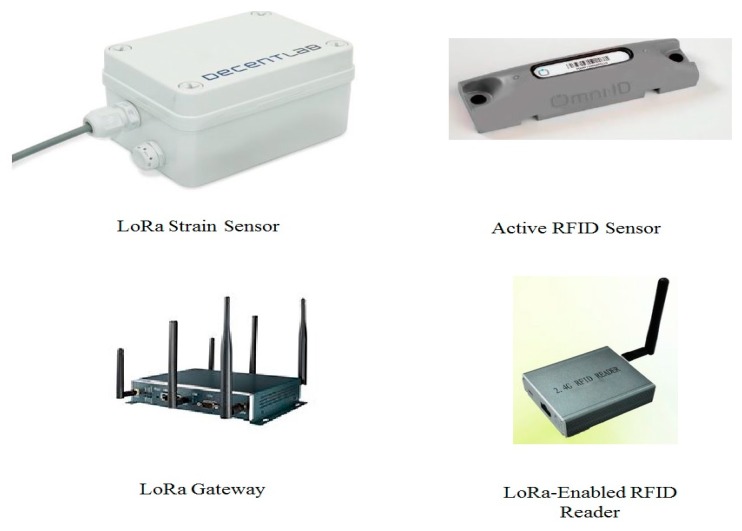
Pictures of the devices used in the proposed method.

The proposed method was tested on a real prefabrication project—a four-level building that is used to test the structural performance of a prefabrication (PC) building during an earthquake. The project is located on a construction site that is a field test laboratory at the China State Construction Engineering Corporation, Beijing, China. Before employing the proposed method on a real project, the sensor network should be examined. The implementation of the proposed method on the PC project was conducted at the construction site. The details of the sensor network test and field test are provided in this section. The sensors and LoRa gateway used in this study are shown in Figure 10.

### 4.1. Sensor Network Test

#### 4.1.1. Sensor Test

**Figure 11:**
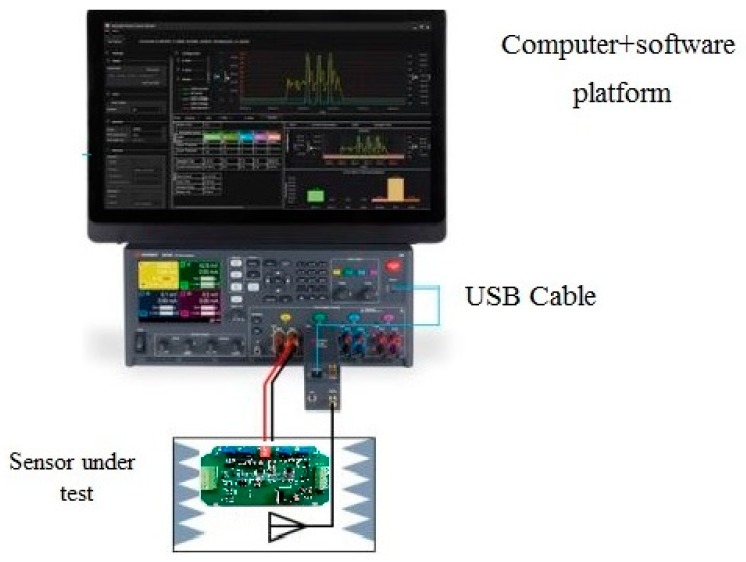
The picture of the sensor test equipment.

**Figure 12:**
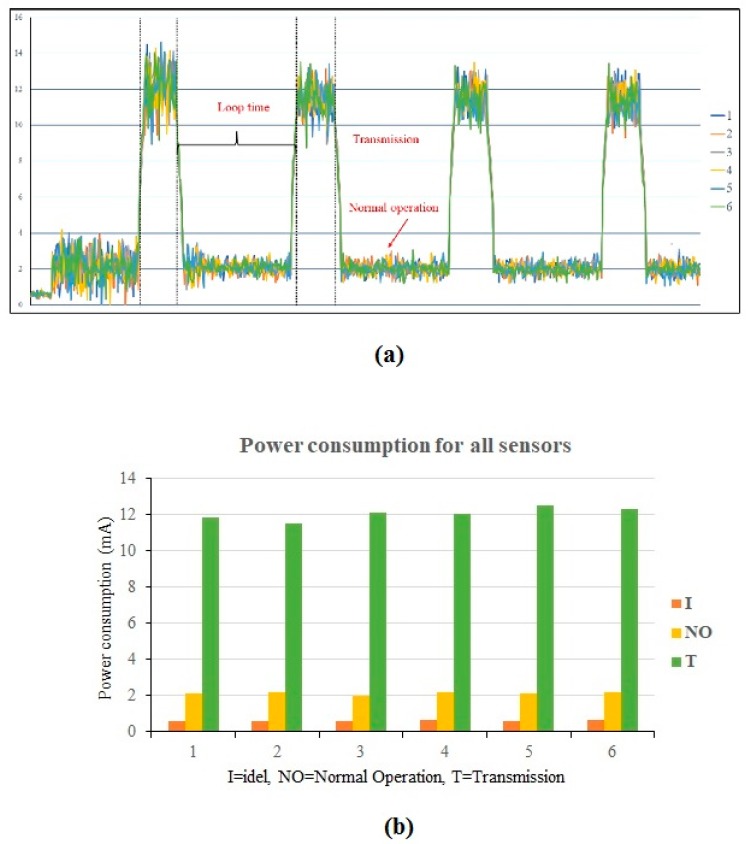
(**a**) Power consumption analysis results of the sensors; (**b**) power consumption analysis results shown in bar chart.

LoRa strain sensors were used in this study to monitor strain level of a PC component. These sensors can detect low-level vibration. The specifications of the sensors are shown in Table 1. Energy consumption is one of the most important criteria to evaluate the feasibility of a wireless sensor network. It is necessary to carry out experiments examining the power consumption of sensors while the radio interface is activating, transmitting, idling, and sleeping. For energy consumption measurements, the data traces are recorded locally, using a Keysight 34972A LXI Data Acquisition Unit, (Santa Rosa, CA, USA) as shown in Figure 11. The unit can be used to record measurements of energy being expended by the sensor every 1–2 s. The measurement process can be run every few minutes, in order to have more accurate estimations of energy consumption. The test results of the six strain sensors (four sensors on a PC slab and two sensors on a PC wall panel) are shown in Figure 12. Based on power consumption analysis, for normal operation, the continuous current I = 2 mA. For data transmission, the current I = 12 mA. The sensor can guarantee a real-time data delivery rate with reasonable energy consumption.

#### 4.1.2. LoRa Network Test

**Figure 13:**
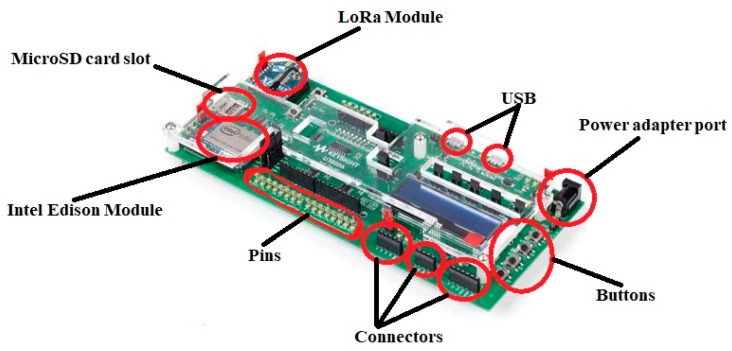
The prototype of the LoRa gateway module.

**Figure 14:**
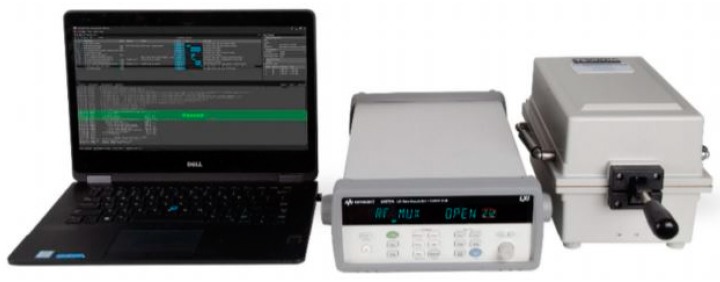
The test equipment for LoRa network.

**Figure 15:**
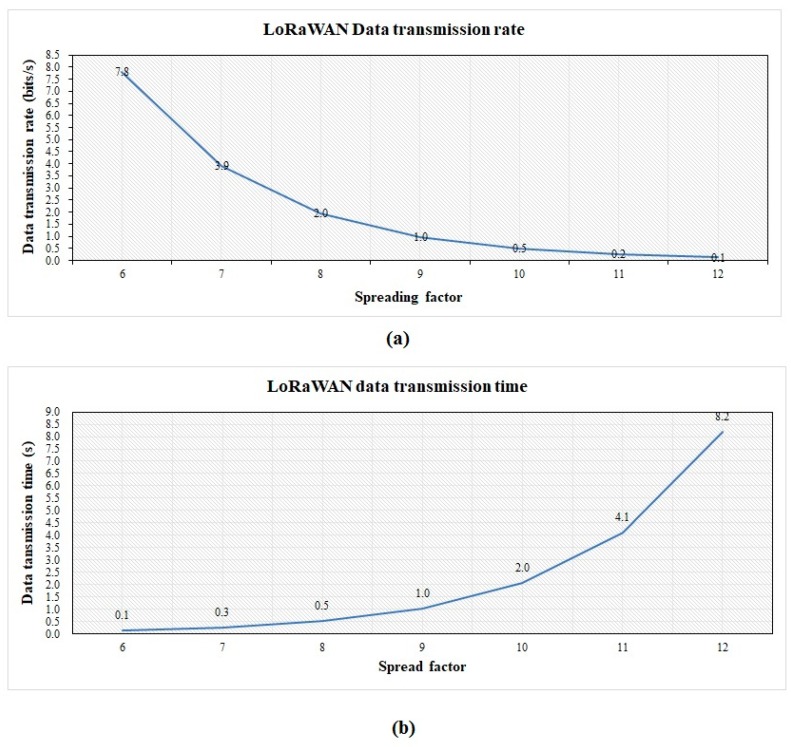
(**a**) Data transmission rate of LoRa network; (**b**) data transmission time of the network.

A LoRa gateway, located at the highest point of a crane on the construction site, collects sensed data. The construction site is a square site 250 m long and 150 m wide. The data collected from the sensors were transmitted to the gateway through LoRaWAN. The LoRa gateway can transmit the data to the on-site computer server and transfer the LoRaWAN protocol to the TCP/IP protocol. Every country has set its own radio frequency to LoRa. In China, LoRa operates in the 470/510 MHz ISM bands, which allows long range coverage, with a bit rate ranging from 0.37 and 46.9 kbps [40]. The spreading factors, such as BW, are important parameters for LoRa communication. The important parameters in LoRa communication include bandwidth, coding rate, spreading factor, and transmission power, as shown in Table 2. The prototype of the used LoRa gateway is displayed in Figure 13. The wireless connectivity between the data sender (sensors) and the data receiver (LoRa gateway) should be examined. The test equipment is shown in Figure 14. The sensor and the gateway were placed inside the shield box for testing the signal transmission. The results are displayed in Figure 15.

### 4.2. Field Test

The dynamic nature of a construction site, and the existence of unpredict events and uncertainties, make it a challenging task to accurately location-tracking and condition-monitoring of a PC component on construction site. A wireless sensor network was employed to obtain real-time data. Upon completion of the configuration of the proposed method, the field test of the proposed method was conducted. The proposed method was applied to a PC building project to validate its effectiveness.

#### 4.2.1. Location of a Prefabrication (PC) Component

A prefabrication (PC) component that enters the jobsite would be registered. In this case, an active RFID sensor was located in each PC component, so that it becomes intelligent and could be uniquely identified and tracked. The properties of the RFID sensor are displayed in Table 3. Three LoRa-enabled RFID readers were located at the same height on the construction site. Based on the RFID technology and triangulation technique, the PC component can be located on the construction site. The PC component would be defined as an IfcBuildingElement. The four-story PC building project on the construction site had four different zones: A (ground floor), B (first floor), C (second floor), and D (third floor), which are modelled in IFC using IfcZone. Each RFID reader was assigned to a zone, using IfcRelAssigns. Incorporating the project data into IFC schema enabled them to be easily integrated into the BIM model.

**Figure 16:**
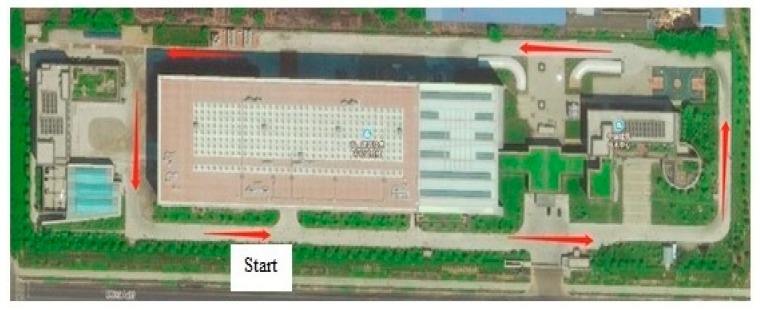
An aerial photograph of the construction site.

**Figure 17:**
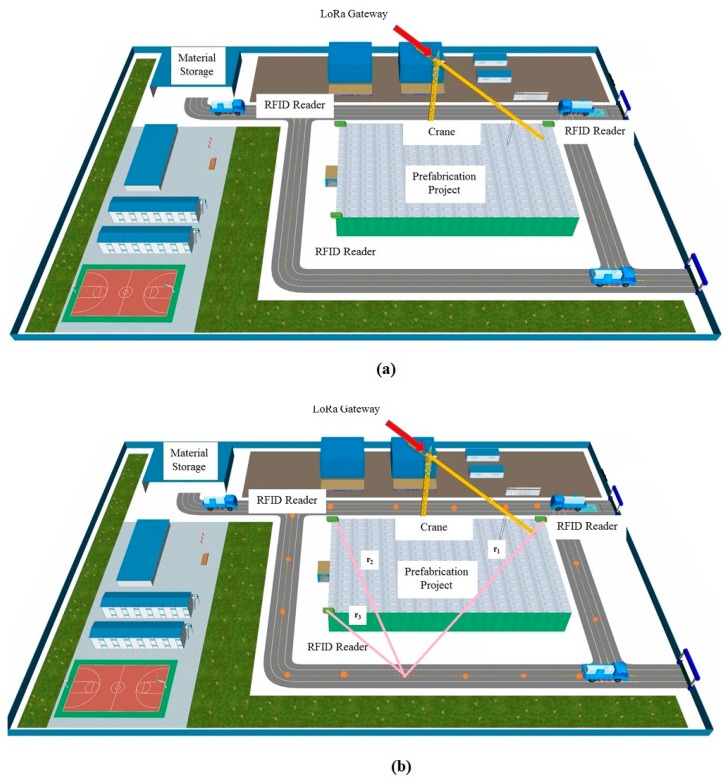
(**a**) The BIM model of the construction site; (**b**) the 16 pre-set locations in the BIM model.

**Figure 18:**
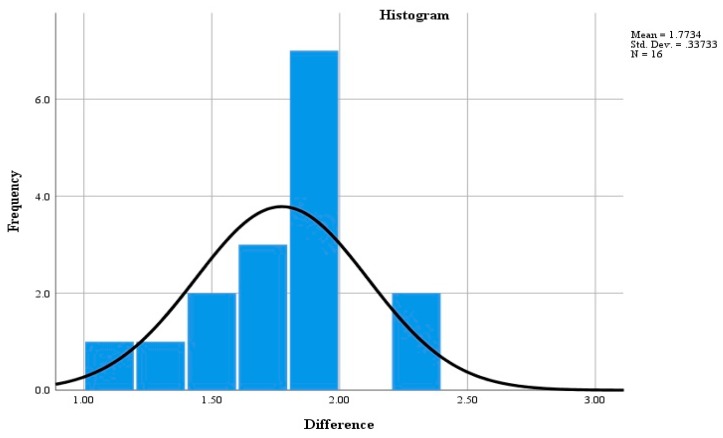
The normal analysis results.

To evaluate the accuracy of the proposed method for locating a PC component on construction site, a test was carried out on the construction site. The picture of the construction site is shown in Figure 16. A BIM model of a construction site was developed, as shown in Figure 17a. Some locations were pre-set and identified in the BIM model, and their coordinates have been saved in the BIM model. This study set 16 pre-set locations, and the coordinates of all the 16 locations were saved in BIM model, as shown in Figure 17b. The actual distances between each pre-set location and the three RFID readers are also saved in BIM model. Prior to the test, a RFID sensor was screwed on the surface of the PC component. During the test, a PC component was transported by a truck, and the truck stopped at each of the pre-set locations to let the three RFID readers obtain the signal. Then the triangulation technique was used to calculate the location of the PC component. The calculated location was saved in the BIM model. Also, the distance between the PC component and the reader is calculated based on Equation (1). The distances between the PC component and the three RFID readers were calculated at each pre-set location. Once the distances of all the 16 locations were calculated, the truck transported the PC component to the material storage area, completing the test. The difference between the actual location and the calculated location of the PC component at *j*-th pre-set location was obtained based on Equation (3). Next, a normal analysis was performed to compare the difference between the coordinates of the actual locations and the calculated coordinates. The results of the statistical analysis are shown in Figure 18. The errors are normally distributed at difference between 1.5 m and 2 m, with 95% accuracy level. The results are acceptable. Therefore, the proposed system can locate a PC component accurately at a construction site:

(3) $$D_{j}=\frac{{\left(r_{j,1}-r_{j,1}^{\prime }\right)}^{2}+{\left(r_{j,2}-r_{j,2}^{\prime }\right)}^{2}+{\left(r_{j,3}-r_{j,3}^{\prime }\right)}^{2}}{3}$$

where $D_{j}$ is the difference between the actual location and measured location of the PC component at the *j*-th pre-set location, *j* = 1,2,..,16; $r_{j,1}$, $r_{j,2}$, $r_{j,\;3}$ is the actual distance between the *j*-th pre-set location and the three RFID readers, respectively; $r_{j,i}^{\prime }$ is the distance calculated based on the Equation (1); *i* = 1, 2, 3, indicating the three different readers on construction site.

#### 4.2.2. Monitoring Results of Strain Level

**Figure 19:**
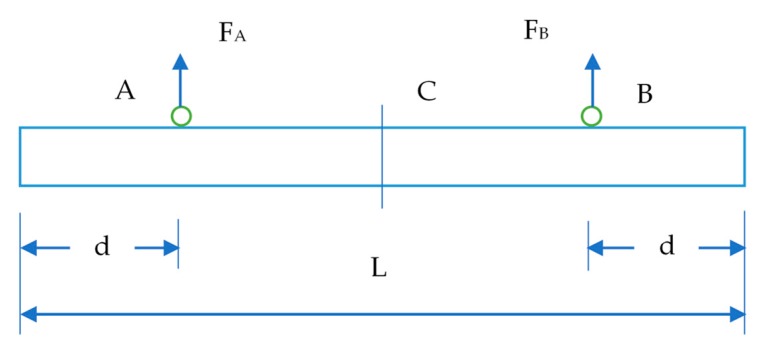
The mechanical model of setting lifting points for a PC wall.

During installation, the distribution of force in the PC components is significantly different from the distribution after loading. A significant redistribution of internal forces may occur during installation. Monitoring the strain level of a PC component is an effective way to evaluate its structural performance. Hence, the strain level of a PC component should be continuously monitored during installation process. In this study, a PC slab and a PC wall panel, which are considered as important PC components for the project, were selected to monitor their structural performance. Strain gauges containing a vibrating wire were attached on the lifting points of a PC component. Hence, the physical locations of lifting points on a PC component should be first determined based on the minimum moment method. When hosting a PC wall component on the construction site, a negative moment is generated at the lifting points, due to the weight of the PC component. Simultaneously, a positive moment is generated at the central point between the two lifting points. According to the principle of minimum moment method, the lifting point should be at the point where the negative moment is equal to the positive moment. The mechanical model of setting lifting points for a PC wall is shown in Figure 19.

Negative moment at point A:

(4) $$M_{A}=\frac{qd^{2}}{2}$$

Positive moment at the central point C:

(5) $$M_{c}=\frac{qL^{2}}{8}-F_{A}\left(\frac{L}{2}-d\right)$$

where

$$F_{A}=qd$$

In order to have the minimum moment, the positive moment must be equal to the negative moment. Hence, the sum of the moments is zero, so M_C_ = −M_A_, and M_C_ + M_A_ = 0. Then, d = 0.207L. Hence, d = 0.207L is the optimum point where the moment is nearly zero. L is the width of the PC wall.

**Figure 20:**
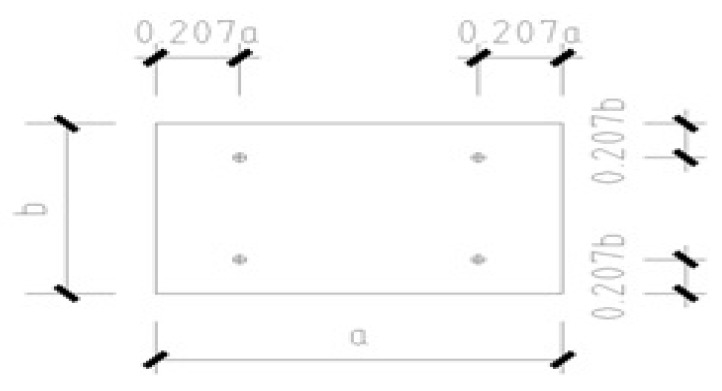
The lifting points of a PC slab.

The minimum moment method is also applicable to the hosting of a PC slab. However, the PC slab has four lifting points. The lifting points should be determined on both the long side and the short side of the slab. Hence, the lifting points are set at d = 0.207a on both ends of the long side and at d = 0.207b on both ends of the short side, where a is the length of the long side and b is the length of the short side, as shown in Figure 20.

**Figure 21:**
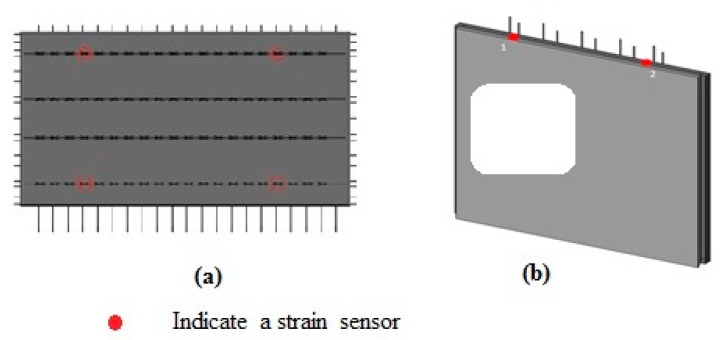
(**a**) BIM model of a PC slab; (**b**) BIM model of a PC wall panel.

The PC slab and PC wall panel are shown in Figure 21a,b, respectively. The positions of the strain sensors located on the PC slab and PC wall panel are also shown in Figure 21a,b, respectively. The PC slab includes four strain sensors that are located at the lifting points. The PC wall has two sensors on top, at the lifting points of the PC wall. The strain sensors are bonded on the PC components using an epoxy resin adhesive.

**Figure 22:**
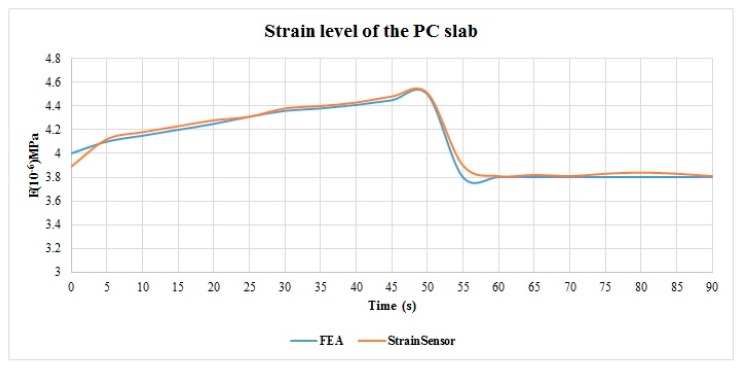
The strain level results of a PC slab obtained from FEA analysis and sensor monitoring.

**Figure 23:**
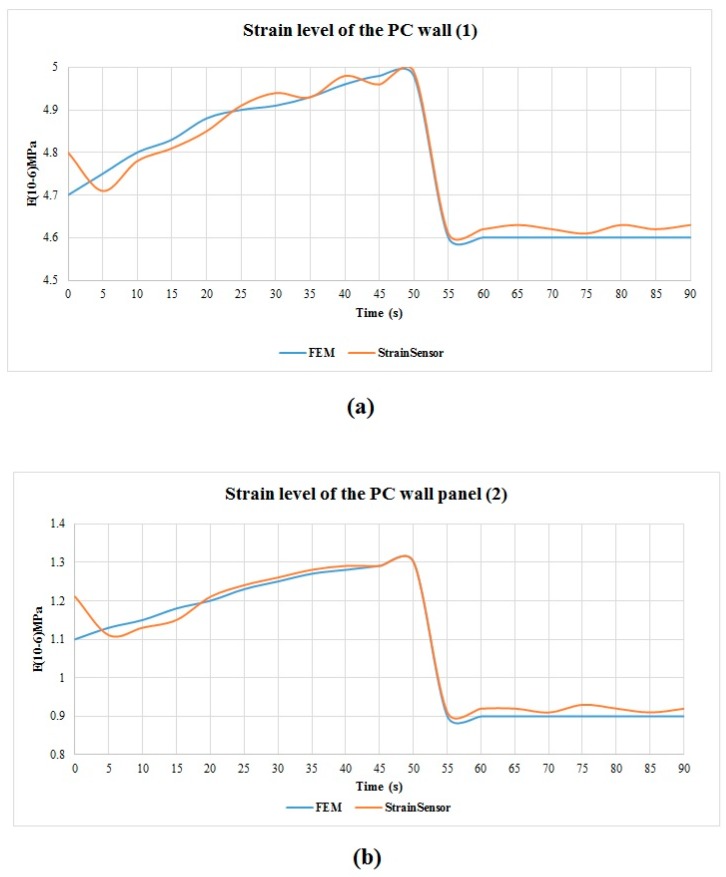
(**a**) The strain level results of lifting point 1 on a PC wall panel obtained from FEA analysis and sensor monitoring; (**b**) The strain level results of lifting point 2 on a PC wall panel obtained from FEA analysis and sensor monitoring.

To validate the reliability of the monitoring data, they were compared with the Finite Element Analysis (FEA) results. The strain values of the four lifting points on the PC slab are same, while the strain values of the two lifting points on the PC wall panel are different. The FEA results and sensor monitoring results of the strain level of the slab are shown in Figure 22. The results of the PC wall panel are shown in Figure 23a,b. The difference between the monitoring data and the results of the Finite Element Analysis (FEA) were examined by using a *t*-test. If the *p*-value of the *t*-test is greater than 0.05, indicating there is no significant difference between the monitoring results and FEA results [40,85]. The *t*-test results are shown in Table 4. As the *t*-test results demonstrated, the monitoring data is appropriately consistent with the FEA analysis results. Hence, the monitoring data was reliable. The results can provide a reference for structural assessment, construction, and decision-making.

#### 4.2.3. The Developed Cloud-based BIM Model

**Figure 24:**
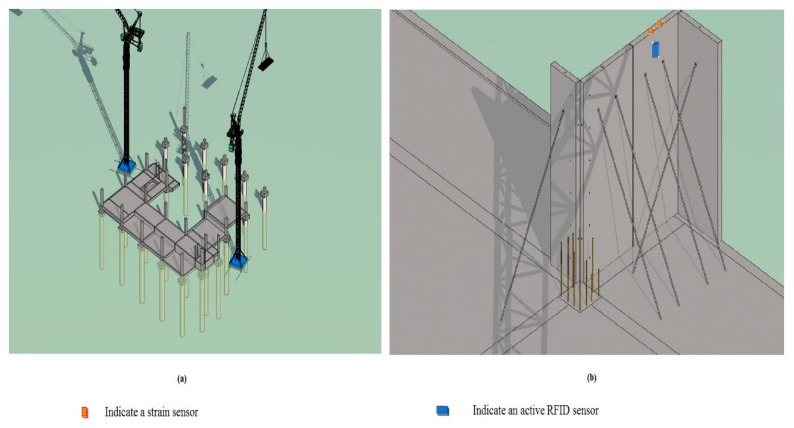
(**a**) BIM model of the PC project; (**b**) BIM model of a PC wall panel holding on site.

Beyond the physical architecture of the LoRa system, a generic, flexible, cloud-based BIM model for analytics and services may create additional value. The cloud-based BIM software, Autodesk BIM 360^TM^ Field, was customized for this study so that it could present the project information. The BIM model data can be inputted into the BIM 360^TM^ Field application. The project information can be accessed through the BIM 360^TM^ Field interface on a mobile device. The BIM model can be completed and updated in Naviswork. Then the BIM model was uploaded to the BIM 360^TM^ Field, where objects were created and linked via Map Model to the relevant object categories. This allowed the BIM model to be synchronized and updated. The sensor data were first transferred to the on-site computer server for preprocessing before further analysis. Sensor data was saved in IFC schema containing three parameters (*ID*, *Sensor_Name*, and *Sensor_Value*). The time-series data, obtained from sensor recordings, is stored in a well-structured database (SQL server database, Microsoft Access) and can be queried using Structured Query Language (SQL). It is important to link the physical sensors to virtual sensors in the BIM model. The Global Unique Identifier (GUID) can be used to define the relationship between physical sensors and virtual objects. This linkage was performed by attaching the ID of a physical sensor to the corresponding virtual sensor in the BIM. Users can log into the corresponding services to access the project information. Moreover, during the installation of the PC project, substantial real-time data is gathered, thereby enabling the BIM model to offer multiple dimensional services and real-time data analysis. The virtual BIM model of the construction site is shown in Figure 24.

## 5. Discussion and Conclusions

In this study, an innovative method was proposed for obtaining real-time information of PC components on site and developing a cloud-based BIM model for the prefabrication project. This method, which integrates cloud-based BIM, RFID, and LoRa technologies, produces a more automatic, systematic, and intelligent system for construction site management, which benefits project management. In order to validate the effectiveness of the proposed method, it was implemented on a real PC project. Before implementation of the field test, the performance of the sensor network was evaluated. The sensor network, when combined with LoRa technology, can provide reliable real-time information, even though errors occasionally occur. In this case study, the proposed method successfully collects and transfers the required data. Even with on-site obstruction and noise, the data could still be successfully transmitted to the host computer. Moreover, the results indicate that the sensor network can obtain and transmit data with reasonable power consumption. Additionally, the results validate the ability of LoRa technology to work collaboratively with sensor networks.

The proposed method suggests a real-time material tracking system based on RFID technology, which can be used in prefabrication projects without interrupting the on-site work process. Being able to provide real time information of PC components on site offers both theoretical and practical advantages. From a theoretical perspective, it becomes possible to use digital methods to obtain real-time information of the PC component on site. From a practical viewpoint, location identification of the PC components is useful for improving project performance. The proposed method provides a promising solution for advanced monitoring of structural health for PC components by offering real-time strain level of the PC components during installation. The monitoring data provides a reference to industry professionals, which allows them to better understand the structural performance of the PC components during installation process. Also, real-time data can be uploaded and integrated to the BIM model for information sharing. The developed cloud-based BIM model allows users to access building information with mobile devices from any location. Adoption of cloud-based data storage allows the user to easily publish and share monitoring data.

The proposed method incorporates IoT technologies and cloud-based BIM model can work effectively and sustainably, which directly supports project management decision-making and improves project performance. It benefits users in several ways. Firstly, the method allows all data to be combined, categorized, and analysed automatically, which reduces the costs associated with manual, error-prone processes. Secondly, it facilitates decision-making process, and ultimately improves project performance. Finally, the method incorporates LoRa technology in site management, which provides a new clue for improving informatization and automation in construction. In order to obtain a reliable method, the proposed method should be applied to more projects. More advanced techniques and technologies can be incorporated into the system to improve its efficiency and effectiveness. For example, edge computing can be introduced to provide a flexible and extensible network architecture. Future studies will focus on two areas. First, 3D printing technology and some new materials will be used to produce peculiar sensors for specific purpose of research projects. Finally, the method can be customized to be used in building operation and maintenance.

## Figures and Tables

**Table 1:** The properties of the LoRa strain sensor.

Parameters	Figures
Range	−45–90 μm
Base frequency	16,500 Hz ± 500 Hz
Signal nominal	12,000–18,000 Hz
Temperature drift	± 0.25 Hz/k

**Table 2:** The properties of LoRaWAN.

Parameter	LoRa Values
Spreading factor	2^7^ to 2^12^
Channel Bandwidth	125 to 500 kHz
Uplink data rate	29–50 kbps
Downlink data rate	27–50 kbps
Efficiency (b/s Hz)	0.12
Doppler sensitivity	Up to 40 ppm
Link budget	156 dB

**Table 3:** The properties of the active RFID tag.

Parameter	Values
Size (mm)	120 × 36 × 30
Weight (g)	92
Battery length	Up to 5 years
Frequency range	433 MHz
Read range	Up to 400 m
RFID protocol	IEEE 802.15.4
Operating temperature	−40 °C–60 °C

**Table 4:** The t-test results of the strain values.

PC Components	*t*-Value	*p*-Value
PC slab	0.749	0.463
PC wall (1)	1.569	0.134
PC Wall (2)	1.871	0.078

## Abbreviations

AEC	Architectural, Engineering, and Construction
AoA	Angle of Arrival
BIM	Building Information Modelling
BMS	Building Management System
BW	Band Width
CR	Coding Rate
FBG	Fiber Bragg Grating
FEA	Finite Element Analysis
GPS	Global Positioning System
GUID	Global Unique Identifier
HTML	Hypertext Markup Language
ID	Identification
IEEE	Institute of Electrical and Electronics Engineers
IFC	Industry Foundation Classes
IMU	Inertial Measurement Unit
IoTs	Internet of Things
ISO	International Organization for Standardization
LoRa	Long Range
LoRaWAN	Long Range Wide Area Network
MVC	Model View Controller
PC	Prefabrication
RFID	Radio Frequency Identification
RSSI	Received Signal Strength Indicator
SF	Spreading Factor
SHM	Structural Health Monitoring
SPAN	Synchronized Position Attitude Navigation
SQL	Structured Query Language
TCP/IP	Transmission Control Protocol/Internet Protocol
ToA	Time of Arrival
USB	Universal Serial Bus
UWB	Ultra-Wide Band
WLAN	Wireless Local Area Network

## References

[1] Li Z., Shen G.Q., Alshawi M. (2014). Measuring the impact of prefabrication on construction waste reduction: An empirical study in China. Resour. Conserv. Recycl..

[2] Wang J., Li Z., Tam V.W. (2015). Identifying best design strategies for construction waste minimization. J. Clean. Prod..

[3] Hong J., Shen G.Q., Mao C., Li Z., Li K. (2016). Life-cycle energy analysis of prefabricated building components: An inputeoutput-based hybrid model. J. Clean. Prod..

[4] Xu G., Li M., Chen C.H., Wei Y. (2018). Cloud asset-enabled integrated IoT platform for lean prefabricated construction. Automat. Construct..

[5] Li X., Shen G.Q., Wu P., Yue T. (2019). Integrating Building Information Modeling and Prefabrication Housing Production. Automat. Construct..

[6] Liu D., Wu Y., Li S., Sun Y. (2016). A real-time monitoring system for lift-thickness control in highway construction. Autom. Constr..

[7] Shi W., Wu C., Wang X. A Prototype Tool of Optimal Wireless Sensor Placement for Structural Health Monitoring. Proceedings of the 25th EG-ICE International Workshop.

[8] Taheri S. (2019). A review on five key sensro for monitoring of concrete structures. Construct. Build. Mater..

[9] Das S., Saha P. (2018). A review of some advanced sensors used for health diagnosis of civil engineering structures. Measurement.

[10] Perera R., Marin R., Ruiz A. (2013). Static–dynamicmulti-scalestructuraldamageidentification in amulti-objectiveframework. J. Sound Vib..

[11] Xu D.L., He W., Li S. (2014). Internet of things in industries: A survey. IEEE Trans. Ind. Inf..

[12] Kochovski P., Stankovski V. (2018). Supporting smart construction with dependable edge computing infrastructures and applications. Automat. Construct..

[13] Rajeswari S., Kannan S., Rajakumar K. A smart agricultural model by integrating IoT, mobile and cloud-based big data analytics. Proceedings of the 2017 International Conference on Intelligent Computing and Contro.

[14] Wang H., Liu D. (2017). Is servitization of construction the inevitable choice of Internet Plus construction?. Front. Eng. Manag..

[15] Valero E., Adán A., Cerrada C. (2015). Evolution of RFID Applications in Construction: A Literature Review. Sensors.

[16] Shin T.H., Chin S., Yoon S.W., Kwon S.W. (2011). A service-oriented integrated information framework for RFID/WSN-based intelligent construction supply chain management. Automat. Construct..

[17] Chen Y., Kamara J.M. (2011). A framework for using mobile computing for information management on construction sites. Automat. Construct..

[18] Wang H., Gluhak A., Meissner S., Tafazolli R. Integration of BIM and live sensing information to monitor building energy performance. Proceedings of the 30th CIB (International Council for Research and Innovation in Building and Construction) International Conference.

[19] Ko H.S., Azambuja M., Lee H.F. (2016). Cloud-based materials tracking system prototype integrated with radio frequency identification tagging technology. Automat. Construct..

[20] Riaz Z., Arslan M., Kiani A.K., Azhar S. (2014). CoSMoS: A BIM and wireless sensor based integrated solution for worker safety in confined spaces. Autom. Constr..

[21] Fang Y., Cho Y.K., Zhang S., Perez E. (2016). Case study of BIM and cloud-enabled real time RFID indoor localization for construction management applications. J. Constr. Eng. Manag..

[22] Montaser A., Bakry I., Alshibani A., Moselhi O. (2012). Estimating productivity of earthmoving operations using spatial technologies. Can. J. Civil Eng..

[23] Diggelen F.V. Indoor GPS theory and implementation. Proceedings of the IEEE Position Location and Navigation Symposium.

[24] Teizer J., Lao D., Sofer M. Rapid automated monitoring of construction site activities using ultra-wideband. Proceedings of the 24th international Symposium on Automation & Robotics in Construction (ISARC).

[25] Aryan A. (2011). Evaluation of Ultra-Wideband Sensing Technology for Position Location in Indoor Construction Environments. Master’s Thesis.

[26] Woo S., Jeong S., Mok E., Xia L., Choi C., Pyeon M., Heo J. (2011). Application of WiFi-Based indoor positioning system for labor tracking at construction sites: A case study in Guangzhou MTR. J. Automat. Construct..

[27] Hightower J., Borriello G. (2001). Location systems for ubiquitous computing. Computer.

[28] Al Nuaimi K., Kamel H. A survey of indoor positioning systems and algorithms. Proceedings of the International Conference on Innovations in Information Technology.

[29] Patwari N., Ash J.N., Kyperountas S., Hero A.O., Moses R.L., Correal N.S. (2005). Locating the nodes: Cooperative localization in wireless sensor networks. Signal. Process. Mag..

[30] Ergen E., Akinci B., East B., Kirby J. (2007). Tracking components and maintenance history within a facility utilizing radio frequency identification technology. J. Comput. Civil. Eng. (ASCE).

[31] Ergen E., Akinci B., Sacks R. (2007). Tracking and locating components in a precast storage yard utilizing radio frequency identification technology and GPS. J. Automat. Construct..

[32] Li H., Chan G., Wong J.K.W., Skitmore M. (2016). Real-time locating systems applications in construction. Autom. Constr..

[33] Kennedy S., Hamilton J., Martell H. Architecture and system performance of SPANNovAtel’s GPS/INS solution. Proceedings of the 2006 IEEE/ION Position, Location, And Navigation Symposium.

[34] Taneja S., Akcamete A., Akinci B., Garrett J.H., Soibelman L., East E.W. (2011). Analysis of three indoor localization technologies for supporting operations and maintenance field tasks. J. Comput. Civ. Eng..

[35] Ding L.Y., Zhou C., Deng Q.X., Luo H.B., Ye X.W., Ni Y.Q., Guo P. (2013). Real-time safety early warning system for cross passage construction in Yangtze riverbed metro tunnel based on the internet of things. Autom. Constr..

[36] Cheng T., Teizer J. (2013). Real-time resource location data collection and visualization technology for construction safety and activity monitoring applications. Autom. Constr..

[37] Aoudia F.A., Gautier M., Magno M., Gentil M.L., Berder O., Benini L. (2018). Long-short range communication network leveraging LoRa™ and wake-up receiver. Microprocess. Microsyst..

[38] LoRa Alliance (2015). LoraWAN™, Specification v1.0.

[39] IEEE (2011). IEEE Standard for Local and Metropolitan Area Networkspart 15.4: Low-Rate Wireless Personal Area Networks (LR-WPANs).

[40] Goursaud C., Gorce J. (2015). Dedicated Networks for IoT:PHY/MAC State of the Art and Challenges.

[41] Pham C., Guo S., Wei G., Xiang Y., Lin X., Lorenz P. (2016). Building Low-Cost Gateways and Devices for Open LoRa IoT Test-Beds. International Conference on Testbeds and Research Infrastructures.

[42] Semtech LoRa modulation basics. https://www.rfwireless-world.com/Terminology/LoRa-modulation-vs-CSS-modulation.html.

[43] Wixted A.J., Kinnaird P., Larijani H., Tait A., Ahmadinia A., Strachan N. Evaluation of LoRa and LoRaWAN for wireless sensor networks. Proceedings of the SENSORS, 2016 IEEE.

[44] Li L., Ren J., Zhu Q. On the application of LoRa LPWAN technology in sailing monitoring system. Proceedings of the 13th Annual Conference on Wireless On-demand Network Systems and Services (WONS).

[45] Vatcharatiansakul N., Tuwanut P., Pornavalai C. Experimental performance evaluation of LoRaWAN: A case study in Bangkok. Proceedings of the 14th International Joint Conference on Computer Science and Software Engineering (JCSSE).

[46] Mikhaylov K., Petäjäjärvi J., Janhunen J. On LoRaWAN scalability: Empirical evaluation of susceptibility to inter-network interference. Proceedings of the Networks and Communications (EuCNC), 2017 European Conference.

[47] Georgiou O., Raza U. (2017). Low power wide area network analysis: Can LoRa scale?. IEEE Wireless Commun. Lett..

[48] Bor M., Roedig U. LoRa Transmission parameter selection. Proceedings of the 13th International Conference on Distributed Computing in Sensor Systems (DCOSS).

[49] Lim J.T., Han Y. (2018). Spreading factor allocation for massive connectivity in LoRa systems. IEEE Commun. Lett..

[50] Babazadeh M. (2019). Edge analytics for anomaly detection in water networks by an Arduino101-LoRa based WSN. ISA Trans..

[51] Hassan W., Føre M., Ulvund J.B., Alfredsen J.A. (2019). Internet of Fish: Integration of acoustic telemetry with LPWAN for efficient real-time monitoring of fish in marine farms. Comput. Electron. Agric..

[52] Addabbo T., Fort A., Mugnaini M., Panzardi E., Pozzebon A., Vignoli V. (2019). A city-scale IoT architecture for monumental structures monitoring. Measurement.

[53] Lynch J.P. (2006). A summary review of wireless sensors and sensor networks for structural health monitoring. Shock Vib. Digest.

[54] Bhalla S., Soh C.K. (2004). High frequency piezoelectric signatures for diagnosis of seismic/blast induced structural damages. NDT&E Int..

[55] Mal A., Ricci F., Banerjee S., Shih F. (2005). A conceptual structural health monitoring system based on vibration and wave propagation. Struct. Health Monit..

[56] Song G., Gu H., Mo Y.L., Hsu T.T.C., Dhonde H. (2007). Concrete structural health monitoring using embedded piezoceramic transducers. Smart Mater. Struct..

[57] Sevillano E., Sun R., Perera R. (2016). Damage Detection Based on Power Dissipation Measured with PZT Sensors through the Combination of Electro-Mechanical Impedances and Guided Waves. Sensors.

[58] Perera R., Pérez A., García-Diéguez M., Zapico-Valle J.L. (2017). ActiveWireless System for Structural Health Monitoring Applications. Sensors.

[59] Ye X.W., Su Y.H., Han J.P. (2014). Structural health monitoring of civil infrastructure using optical fiber sensing technology: A comprehensive review. Sci. World J..

[60] Roussel M., Glisic B., Lau J.M., Fong C.C. (2014). Long-term monitoring of high-rise buildings connected by link bridges. J. Civil. Struct. Health Monit..

[61] Wong K.Y. (2004). Instrumentation and health monitoring of cable supported bridges. Struct. Control. Health Monit..

[62] Jang S., Jo H., Cho S., Mechitov K., Rice J.A., Sim S.H., Jung H.J., Yun C.B., Spencer B.F.J., Agha G. (2010). Structural health monitoring of a cable-stayed bridge using smart sensor technology: Deployment and evaluation. Smart Struct. Syst..

[63] Magalhães F., Cunha A., Caetano E. (2012). Vibration based structural health monitoring of an arch bridge: From automated OMA to damage detection. Mech. Syst. Signal. Process..

[64] Hu X., Wang B., Ji H. (2013). A wireless sensor network-based structural health mon- itoring system for highway bridges. Comput. Aided Civil. Infrastruct. Eng..

[65] Tang Y.C., Li L.J., Feng W.X., Liu F., Zou X.J., Chen M.Y. (2018). Binocular vision measurement and its application in full-field convex deformation of concrete-filled steel tubular columns. Measurement.

[66] Chen M., Tang Y., Zou X., Huang K., Li L., He Y. (2019). High-accuracy multi-camera reconstruction enhanced by adaptive point cloud correction algorithm. Opt. Lasers Eng..

[67] Costin A., Pradhananga N., Teizer J. (2012). Leveraging passive RFID technology for construction resource field mobility and status monitoring in a high-rise renovation project. Automat. Construct..

[68] Guven G., Ergen E., Erberik M.A., Kurc O., Birgönül M.T. (2012). Providing guidance for evacuation during an emergency based on a real-time damage and vulnerability assessment of facilities. Comput. Civil. Eng..

[69] Dong B., O’Neill Z., Li Z. (2014). A BIM-enabled information infrastructure for building energy fault detection and diagnostics. Autom. Constr..

[70] Pasini D., Ventura S.M., Rinaldi S., Bellagente P., Flammini A., Ciribini A.L.C. Exploiting Internet of Things and building information modeling framework for management of cognitive buildings. Proceedings of the 2016 IEEE International Smart Cities Conference.

[71] Alves M., Carreira P., Aguiar Costa A. (2017). BIMSL: A generic approach to the integration of building information models with real-time sensor data. Autom. Constr..

[72] Khalid M.U., Bashir M.K., Newport D., Dastbaz M., Gorse C., Moncaster A. (2017). Development of a building information modelling (BIM)-based real-time data integration system using a building management system (BMS). Building Information Modelling, Building Performance, Design and Smart Construction.

[73] Wang M., Li R., Zhang W. (2018). Application and Mechanics Analysis of Multi-function Construction Platforms in Prefabricated-Concrete Construction. Int. J. Eng. Technol..

[74] Gunawardena T., Ngo T., Mendis P., Alfano J. (2016). Innovative Flexible Structural System Using Prefabricated Modules. J. Archit. Eng..

[75] Kong X., Li J., Laflamme S., Bennett C., Matamoros A. Characterization of a soft elastomeric capacitive strain sensor for fatigue crack monitoring. Proceedings of the SPIE Smart Structures and Materials + Nondestructive Evaluation and Health Monitoring.

[76] Downey A., D’Alessandro A., Laflamme S. (2017). Smart bricks for strain sensing and crack detection in masonry structures. Smart Mater. Struct..

[77] Park G., Rosing T., Todd M.D., Farrar C.R., Hodgkiss W. (2008). Energy harvesting for structural health monitoring sensor networks. J. Infrastruct. Syst..

[78] Liu T., Yang B., Zhang Q. (2017). Health monitoring system developed for Tianjin 117 high-rise building. J. Aerosp. Eng..

[79] Demers D. LoRa Networks in Buildings Reduce Infrastructure Costs. http://iotdesign.embedded-computing.com/articles/lora-networks-in-buildings-reduce-infrastructure-costs/.

[80] International Organization for Standardization (2013). Industry Foundation Classes (IFC) for Data Sharing in the Construction and Facility Management Industries.

[81] International Organization for Standardization (2004). Industrial Automation Systems and Integration—Product Data Representation and Exchange—Part 11: Description Methods: The EXPRESS Language Reference Manual.

[82] IFC (2014). Industry Foundation Classes Release 4.

[83] Motamedi A., Soltani M.M., Setayeshgar S., Hammad A. (2016). Extending IFC to incorporate information of RFID tags attached to building elements. Adv. Eng. Inf..

[84] Johansson M., Roupé M., Bosch-Sijtsema P. (2015). Real-time visualization of building information models (BIM). Automat. Construct..

[85] Lin J., Zhang J., Wen Q., Wang F. Leveraging BIM in Settlement Monitoring and Impact Management for Subway Excavation. Proceedings of the 32nd CIB W78 Conference.

